# Incidence and risk factors for phaeochromocytoma diagnosis in dogs under primary veterinary care in the UK

**DOI:** 10.1371/journal.pone.0332811

**Published:** 2025-10-15

**Authors:** Martin Litviakov, Dan G. O’Neill, Dave C. Brodbelt, Sara Galac, Floryne O. Buishand

**Affiliations:** 1 Department of Clinical Science and Services, Royal Veterinary College, Hatfield, United Kingdom; 2 Department of Pathobiology and Population Sciences, Royal Veterinary College, Hatfield, United Kingdom; 3 Department of Clinical Sciences, Faculty of Veterinary Medicine, Utrecht University, Utrecht, The Netherlands; Long Island University - CW Post Campus: Long Island University, UNITED STATES OF AMERICA

## Abstract

Phaeochromocytoma (PCC) is a tumour arising from the adrenal gland in dogs that can be challenging to diagnose. This study aimed to describe the incidence risk, breed predispositions and other demographic risk factors associated with the diagnosis PCC in dogs receiving primary veterinary care in the UK. All anonymised VetCompass Programme electronic health records from dogs receiving primary veterinary care in the UK during 2019 were included. Demographic risk factor analysis used multivariable logistic regression modelling. Out of a study population of 2,250,741 dogs, 92 were confirmed as PCC cases at any time point. The estimated 2019 incidence risk for PCC diagnosis was 1 per 100,000 dogs. The Soft-Coated Wheaten Terrier, German Pointer and Miniature Schnauzer showed significant breed predispositions for the diagnosis of PCC compared with crossbred dogs. Terrier breeds and breeds predisposed to other endocrine tumours were found to have increased odds of being diagnosed with PCC. Furthermore, neutered males and dogs aged between 9 and under 15 years were also associated with increased odds of PCC diagnosis. This study is the first to describe the epidemiology of PCC in dogs receiving primary veterinary care, providing new information concerning demographic risk factors and helping to improve clinical recognition of PCC for veterinary clinicians. Moreover, results from this study facilitate further research in the possible links of canine PCC with other canine endocrine tumours and the existence of concurrent endocrine neoplasia in dogs. Additionally, the results allow researchers to more robustly define useful PCC study populations for future comparative oncology studies.

## Introduction

Phaeochromocytoma (PCC) is a neuroendocrine tumour described in several species, sharing many similarities between dogs and humans [1,2]. PCC arises from the chromaffin cells which are responsible for producing and storing catecholamines in the adrenal medulla. These tumours can cause unpredictable episodic overproduction of catecholamines such as epinephrine and norepinephrine, leading to non-specific clinical signs such as hypertension, tachycardia, weakness and abdominal pain in dogs, with anxiety, headaches and feelings of panic also being common in humans [3–8]. Human and canine PCC show similar genomic alterations, clinical signs, metastatic frequency and share similar treatment approaches, positioning dogs as a potentially more translationally relevant model than rodents [2,9–11]. Rodent models are constrained by key genomic differences in tumour suppression pathways, particularly involving the succinate dehydrogenase subunit B/D (*SDHB/D*) genes, and require artificial tumour induction, whereas PCC occurs spontaneously in dogs [9,12–16].

PCC is an uncommon clinical diagnosis, with the reported annual incidence in humans ranging from 1.9 to 4.6 cases per million per year [1,17–19]. However, a relatively higher prevalence of PCC in autopsy studies of 0.05% suggests that PCC could be heavily under-diagnosed in humans and result in premature mortality [8,20,21]. Clinical diagnosis of PCC is also considered rare in dogs although to the authors’ knowledge the incidence of canine PCC has not yet been reported [5,6]. However, similarly to the human disease, it is likely that canine PCC is also substantially under-diagnosed because of its non-specific clinical signs that can commonly occur in other cardiovascular, neurological or endocrine diseases. Moreover, formal diagnosis of PCC can be challenging in primary care veterinary practice as the identification of an adrenal mass requires abdominal ultrasonography with a skilled operator, or access to advanced imaging modalities such as computed tomography (CT). It is not uncommon for PCC to be diagnosed incidentally when an adrenal mass is visualised whilst assessing the abdomen for another problem [22,23]. Biochemical testing and measurement of plasma or urine free normetanephrine concentrations are also used in both dogs and humans as means of identifying PCC, but have only been introduced to veterinary medicine over the past 10–15 years and made readily commercially available even more recently [24–28]. However, definitive diagnosis of PCC relies on histopathology and immunohistochemistry [2,29,30].

In humans, up to 24% of PCC are related to hereditary tumour syndromes including Multiple Endocrine Neoplasia type 2 (MEN2), Multiple Endocrine Neoplasia type 3 (MEN3), von-Hippel-Lindau syndrome, neurofibromatosis type 1 and PCC-paraganglioma (PGL) syndrome, which implies that genetic testing for these syndromes should be routine for human PCC patients as they may be predisposed to the occurrence of neoplasia in multiple other endocrine organs [31,32]. However, the veterinary literature is quite limited regarding the existence of equivalent hereditary tumour syndromes in dogs [16,33,34].

Case series and case reports constitute most of the current veterinary literature on canine PCC, with most case series counting fewer than 60 dogs and mainly focusing on the clinical, biochemical, imaging, treatment and pathologic findings of the disease [5,6,35–38]. These case series have not suggested any sex or breed predispositions [5,6]. The reported mean age and median bodyweight at time of diagnosis being 10–12 years and 17 kg respectively, suggesting that medium-sized middle-aged to older dogs are the most represented population [6,11].

Over the last ten years, veterinary epidemiological studies of the frequency and risk factors for neoplasia in dogs and cats are increasingly being published using large primary care datasets, thus providing epidemiological knowledge that could not be obtained from past case series. This ‘big data approach’ has allowed veterinary epidemiological studies to match the study designs and population numbers of human epidemiological studies, which are essential to improving the understanding of rare diseases [39–41]. A major player in the improvement of veterinary epidemiological outputs has been the VetCompass Programme which shares anonymised electronic health records (EHRs) from over 30% of all UK practices and includes over 20 million animals [42]. VetCompass has already supported several publications in veterinary oncological epidemiology, determining the incidence and risk factors for canine insulinoma, osteosarcoma and mammary tumours amongst others [43–45]. With this background, the present study aimed to use VetCompass data to report the annual (2019) prevalence and incidence risk of PCC, as well as demographic risk factors, with an emphasis on breed associations, for dogs under primary veterinary care in the UK. A better understanding of PCC epidemiology not only equips veterinary clinicians to recognise and diagnose PCC earlier, but it could also strengthen the dog’s value as a spontaneous translational model for human PCC research.

## Materials and methods

This study utilised data from 2,250,741 dogs under primary veterinary care in 2019, collected from veterinary practices across the UK participating in the VetCompass Programme, which compiles anonymised EHRs for epidemiological research [42]. Dogs were considered under veterinary care if they had at least one EHR entry (clinical note, treatment, or bodyweight record) in 2019. Available data fields included a unique animal ID, species, breed, date of birth, sex, neuter status, as well as clinical notes, treatment records, and bodyweight entries, each with corresponding dates. For this study, cohort EHR data were reviewed up to August 31, 2024 for dogs with at least one EHR recorded in 2019.

A retrospective cohort study design was employed to estimate the available-EHR prevalence (i.e., cases meeting the PCC case definition up to August 31, 2024), the annual prevalence in 2019, and the incidence risk for PCC diagnosis in 2019. The study also examined the relationship between demographic risk factors and the likelihood of a PCC diagnosis. And initial screening indicated a crude prevalence of PCC at 0.01%, and based on this, a sample size of at least 150,747 dogs was calculated to estimate this frequency with a 0.005% margin of error at 95% confidence, assuming an 8-million-dog population in the UK [46]. Ethical approval was granted by the RVC Social Science Research Ethical Review Board (reference: SR2018−1652).

A dog was classified as a PCC case if it met at least one of the following criteria:

1. Evidence of a recorded final diagnosis of PCC in available EHRs.
2. Histopathological confirmation of PCC in the available clinical records.
3. Evidence in all available clinical records of a plasma free normetanephrine concentration >3.6 nmol/L, or metanephrine concentration >2.5 nmol/L, or urinary normetanephrine:creatinine >300 nmoL/L, combined with the demonstration of an adrenal mass on ultrasound or computed tomography.
4. At least one recorded prescription of phenoxybenzamine, combined with the demonstration of an adrenal mass or caval thrombus on ultrasound or CT.

Dogs that were initially diagnosed with PCC but where this diagnosis was later ruled out were excluded. Case-finding involved a two-step process. Candidate PCC cases were initially identified through a search of the clinical notes using specific search terms: ‘phaeo*’, ‘feoc*’, ‘pheoc*’, ‘normetane*’, ‘metane*’, ‘phenox*’, ‘adrenalect*’, ‘caval throm*’, and ‘caval inv*’ and the treatment fields using the search terms: ‘phenox*’ and ‘adrenalect*’. Secondly, EHRs of all candidate cases were manually reviewed to determine eligibility for inclusion. A random sample of 500,000 non-PCC cases out of dogs not identified as candidate cases was used as controls in risk factor analysis.

Data cleaning was performed in Excel (Microsoft Office Excel 2016), and statistical analyses were conducted using SPSS^®^Statistics version 30 (IBM^®^). Breed details recorded by the participating practices underwent cleaning and were mapped to a VetCompass breed list, which was based and expanded upon from the VeNom Coding breed list, encompassing both acknowledged purebred breeds as well as designer breed terms [47]. A breed purity variable (termed *Purebred*) was used to classify dogs as follows: recognised breeds were labelled ‘purebred’, dogs with contrived names combining two or more purebred terms were categorised as ‘designer’ crossbreeds (intentionally bred crosses) and dogs recorded as mixed breed without a specific name were classified as ‘crossbreed’ [48]. A *Breed* variable included individual pure breeds and designer hybrids with at least two PCC cases, while all other breeds were grouped together, along with a separate grouping for general crossbreed dogs. This classification aimed to enhance statistical power of the analyses for individual breeds [49]. A *terrier* variable classified breeds as ‘terrier’ or ‘non-terrier’ based on a combined grouping from the Kennel Club and VeNom Coding Group [45,47,48]. Variables of breeds predisposed for various types of endocrine cancer were used as previously described by Kraai *et al.* (2025) [45].

Sex and neuter status were combined into one variable for the risk factor analysis based on the final EHR record. Median adult bodyweight was calculated using all bodyweights recorded after 18 months of age for each dog and categorised as: < 10.0 kg, 10.0 to < 20.0 kg, 20.0 to < 30.0 kg and ≥ 30.0 kg. Age at first diagnosis was based on date of birth and date of first PCC diagnosis. Age of the non-PCC cases was defined at December 31, 2019. Age categories were classified into: < 6 years, 6 – < 9 years, 9 – < 12 years, 12 – < 15 years, ≥ 15 years and unrecorded. Normally distributed continuous variables were summarised as mean (standard deviation [SD]) while non-normally distributed data were presented as median (interquartile range [IQR] and range).

The available-EHR prevalence of PCC diagnosis was calculated by dividing the total number of diagnosed cases at any time up to August 31, 2024, by the total number of dogs in the study. The one-year period prevalence for 2019 included all cases diagnosed up to December 31, 2019, divided by the same denominator population. Annual incidence risk for 2019 was defined as the number of newly diagnosed cases that year divided by the denominator population. Breed available-EHR prevalence was calculated similarly, by dividing the number of PCC cases of a breed within all available EHRs by the number of dogs of that breed within the denominator population. Confidence intervals (CIs) were calculated using standard errors, based on an approximation to the binomial distribution.

Risk factor analysis included all cases diagnosed up to August 31, 2024 (i.e., available-EHR risk) to maximise statistical power. Binary logistic regression modelling was used to assess univariable associations between potential risk factors (sex/neuter status, breed, purebred status, median adult bodyweight, age, median adult bodyweight in relation to the median for the sex/breed, terrier breed status, and known breed predisposition to other endocrine cancers) and the outcome of having a PCC diagnosis at any point up to August 31, 2024 among dogs under primary veterinary care in 2019. Risk factors with an inflated cut-off *P*-value < 0.2 in univariable screening analyses were included in multivariable analysis, following the standardised methodology of previously published VetCompass studies [43–45,50,51]. This purposeful selection of covariates with an inflated cut-off *P-*value < 0.2 in univariable analysis has been demonstrated to be superior to the use of more traditional *P*-value cut-off points such as 0.05 or 0.1, as those lower cut-offs can fail in identifying variables known to be important [52]. Median adult bodyweight was considered a defining characteristic of individual breeds and was therefore excluded from initial breed-focused multivariable modelling [48]. Other variables directly derived from the *Breed* variable (e.g., *Terrier, Breed predisposed for one of multiple types of endocrine cancer*) were similarly not considered in initial breed-focused multivariable modelling, but individually replaced the *Breed* variable in the final breed-focused multivariable model to test their effects after taking account of the other variables in that model [53]. Model fit and discriminatory performance of the final breed multivariable model were evaluated using the area under the receiver operating characteristic (ROC) curve and the Hosmer-Lemeshow test. Variables were retained in the final multivariable logistic regression model if they met the threshold for statistical significance (*P* < 0.05).

## Results

### Demography

**Fig 1:**
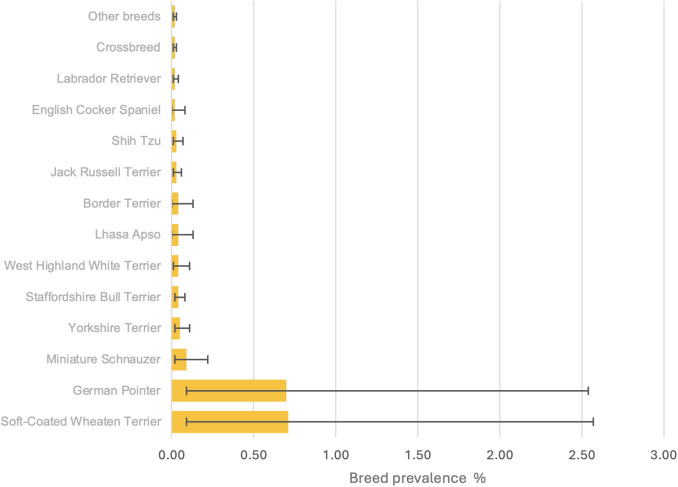
Available-EHR breed prevalence of phaeochromocytoma diagnosis in dog breeds with ≥ 2 phaeochromocytoma cases (*n *= 69) under primary veterinary care at practices in the VetCompass Programme in the UK in 2019. The error bars show the 95% confidence interval.

Among the study population of 2,250,741 dogs under primary veterinary care in 2019, 1,222 (0.05%) candidate PCC cases were identified. Following manual review, 92/1,222 (7.53%) candidates were confirmed as PCC cases that met the inclusion criteria at any time in the available EHRs up to August 31, 2024. The dates of first recorded PCC diagnoses for the 92 cases were between April 1, 2016 and August 31, 2024. The available-EHR prevalence for PCC diagnosis was 4 per 100,000 dogs. Of the 92 cases, 32 cases were diagnosed on or before December 31, 2019, corresponding to a 2019 annual prevalence of 1 per 100,000 dogs. Eighteen cases were newly diagnosed in 2019, yielding an annual (2019) incidence risk for PCC diagnosis of 1 per 100,000 dogs. Of breeds with ≥ 2 PCC cases, the breeds with the highest available-EHR prevalence were Soft-Coated Wheaten Terrier (*n* = 2, 0.71%, 95% CI 0.09–2.57%), German Pointer (*n* = 2, 0.09%, 95% CI 0.09–2.54%), Miniature Schnauzer (*n* = 4, 0.09%, 95% CI 0.02–0.22%) and Yorkshire Terrier (*n* = 6, 0.05%, 95% CI 0.02–0.11%) (Fig 1).

Of the 92 PCC cases diagnosed to August 31, 2024, 65 (70.7%) were male (with 75.4% neutered) and 27 (29.4%) were female (with 77.8% neutered). Age at first diagnosis was available for 76/92 (82.6%) dogs. The median age at first PCC diagnosis was 10.6 years (IQR 9.3–12.3, range 5.6–14.4). Median adult bodyweight was available for 82/92 (89.1%) cases. The median of the median adult bodyweight was 12.8 kg (IQR 9.1–23.3, range 4.6–68.9). The most frequently diagnosed breeds with PCC were crossbreed (**n* *= 22), Staffordshire Bull Terrier (**n* *= 8), and Jack Russell Terrier (**n* *= 7) (Table 1).

**Table 1:** Descriptive statistics and univariable logistic regression results evaluating breed as a risk factor for phaeochromocytoma diagnosis in 92 dogs at any date in all available EHRs compared with a randomly selected control group of 500,000 dogs under UK primary veterinary care in 2019, as recorded in the VetCompass database.

Breed	Phaeochromocytoma cases*, n* (%)	Non-phaeochromocytoma cases, *n* (%)	Odds ratio	95% CI*	Category *P*-value	Variable *P*-value
Crossbreed	22 (23.91)	119,096 (23.82)	Ref**			**<0.001**
Soft-Coated Wheaten Terrier	2 (2.17)	281 (0.06)	38.53	9.02-164.63	**<0.001**	
German Pointer	2 (2.17)	285 (0.06)	37.99	8.89-162.31	**<0.001**	
Miniature Schnauzer	4 (4.35)	4,682 (0.94)	4.63	1.59-13.43	**0.005**	
Yorkshire Terrier	6 (6.52)	11,671 (2.33)	2.78	1.13-6.87	**0.026**	
Staffordshire Bull Terrier	8 (8.70)	20,746 (4.15)	2.09	0.93-4.69	0.075	
West Highland White Terrier	3 (3.26)	7,866 (1.57)	2.07	0.62-6.90	0.239	
Lhasa Apso	2 (2.17)	5,473 (1.10)	1.98	0.47-8.42	0.356	
Border Terrier	2 (2.17)	5,535 (1.11)	1.96	0.46-8.32	0.364	
Jack Russell Terrier	7 (7.61)	22,612 (4.52)	1.68	0.72-3.92	0.234	
Shih Tzu	4 (4.35)	15,028 (3.01)	1.44	0.50-4.18	0.502	
English Cocker Spaniel	2 (2.17)	9,483 (1.90)	1.14	0.67-4.86	0.858	
Labrador Retriever	5 (5.43)	34,235 (6.85)	0.79	0.30-2.09	0.635	
Other breeds	23 (25.00)	243,007 (48.60)	0.51	0.29-0.92	**0.025**	
Total	92 (100.00)	500,000 (100.00)				

Column percentages are presented in brackets. *CI, confidence interval. **Ref, reference category used as the baseline for comparison with other groups. Statistically significant results (*P* < 0.05) are highlighted in bold.

Among the random sample of 500,000 non-PCC cases, 258,026 (51.6%) were male (with 43.0% neutered), 237,611 (47.5%) were female (with 44.6% neutered) and the sex was unrecorded in 4,363 cases (0.9%). The median age at December 31, 2019 was available for 496,135/500,000 (99.2%), with a median age of 5.2 years (IQR 2.2–9.0, range 0.0–24.8). The median adult bodyweight was available for 342,535/500,000 (68.5%) dogs, with a median of the median adult bodyweight of 13.7 kg (IQR 8.4–24.4, range 1.5–106.0). The most frequently represented breeds in the non-PCC cases group were crossbreed (**n* *= 119,096), Labrador Retriever (**n* *= 34,325), and Jack Russell Terrier (**n* *= 22,612) (Table 1).

### Risk factor analysis

Univariable binary logistic regression identified 13 variables meeting the inflated cut-off (**P* *< 0.20) of univariable screening for a liberal association with PCC diagnosis recorded at any time up to August 31, 2024: sex-neuter status, age, median adult body weight, breed, median adult bodyweight in relation to the median for the sex/breed, terrier breed status, breed predisposed for mammary gland cancer, breed predisposed for pituitary/cortisol-secreting adrenal cancer, breed predisposed for pituitary/cortisol-secreting adrenal cancer and/or insulinoma, breed predisposed for parathyroid and/or thyroid and/or pituitary/cortisol-secreting adrenal cancer, breed predisposed for parathyroid and/or thyroid and/or pituitary/cortisol-secreting adrenal cancer and/or insulinoma, breed predisposed for reproductive endocrine organ cancer and breed predisposed for one or multiple types of endocrine cancer (Tables 1 and 2).

**Table 2:** Descriptive statistics and univariable logistic regression results evaluating demographic risk factors for phaeochromocytoma diagnosis in 92 dogs at any date in all available EHRs compared with a randomly selected control group of 500,000 dogs under UK primary veterinary care in 2019, as recorded in the VetCompass database.

Variable	Phaeochromocytoma cases*, n* (%)	Non-phaeochromocytoma cases, *n* (%)	Odds ratio	95% CI*	Category *P*-value	Variable *P*-value
Sex-neuter						**<0.001**
Female entire	6 (6.52)	131,611 (26.32)	Ref**			
Female neutered	21 (22.83)	106,000 (21.20)	4.35	1.75-10.77	**0.002**	
Male entire	49 (53.26)	147,198 (29.44)	2.38	0.93-6.09	0.070	
Male neutered	16 (17.39)	110,828 (22.17)	9.70	4.15-22.64	**<0.001**	
Unrecorded	0 (0)	4,363 (0.87)	0	0	0.985	
Age at time of diagnosis (phaeochromocytoma cases) and age at December 31 2019 (non-phaeochromocytoma cases)						**<0.001**
0 – < 6	1 (1.09)	276,619 (55.32)	0.03	0.00-0.19	**<0.001**	
6 – < 9	14 (15.22)	96,646 (19.32)	Ref			
9 – < 12	46 (50.00)	71,440 (14.29)	4.45	2.44-8.09	**<0.001**	
12 – < 15	29 (31.52)	40,442 (8.09)	4.95	2.62-9.37	**<0.001**	
≥15	1 (1.09)	10,987 (2.20)	0.63	0.08-4.78	0.653	
Unrecorded	1 (1.09)	3,866 (0.77)	1.79	0.24-13.58	0.575	
Median adult bodyweight						**<0.001**
<10 kg	27 (29.35)	118,594 (23.72)	1.65	0.72-3.80	0.236	
10 – < 20 kg	28 (30.43)	106,103 (21.22)	1.92	0.84-4.39	0.124	
20 – < 30 kg	19 (20.65)	66,991 (13.40)	2.06	0.87-4.90	0.102	
≥30 kg	7 (7.61)	50,847 (10.17)	Ref			
Unrecorded	11 (11.96)	157,465 (31.49)	0.51	0.20-1.31	0.161	
Purebred						0.565
Crossbreed	22 (23.91)	119,096 (23.82)	Ref			
Designer	4 (4.35)	33,201 (6.64)	0.65	0.23-1.89	0.432	
Purebred	66 (71.74)	344,711 (68.94)	1.04	0.64-1.68	0.884	
Unrecorded	0 (0.0)	2,992 (0.60)	0	0	0.986	
Median adult bodyweight in relation to the median for the sex/breed						**<0.001**
At or below median adult bodyweight for the sex/breed	33 (35.87)	171,519 (34.30)	Ref			
Above median adult bodyweight for the sex/breed	48 (52.17)	169,624 (33.92)	1.47	0.94-2.29	0.088	
Unrecorded	11 (11.96)	158,857 (31.77)	0.36	0.18-0.71	**<0.001**	
Terrier						**<0.001**
Non-terrier	66 (71.74)	435,007 (87.00)	Ref			
Terrier	26 (28.26)	64,993 (13.00)	2.64	1.68-4.15	**<0.001**	
Breed predisposed for mammary gland cancer						**0.019**
Not predisposed	54 (58.70)	349,891 (69.98)	Ref			
Predisposed	38 (41.30)	150,109 (30.02)	1.64	1.08-2.48	**0.019**	
Breed predisposed for parathyroid cancer						0.554
Not predisposed	86 (93.48)	458,892 (91.78)	Ref			
Predisposed	6 (6.52)	41,108 (8.22)	0.78	0.34-1.78	0.554	
Breed predisposed for thyroid cancer						0.634
Not predisposed	90 (97.83)	484,849 (96.97)	Ref			
Predisposed	2 (2.17)	15,151 (3.03)	0.71	0.18-2.89	0.634	
Breed predisposed for insulinoma						0.416
Not predisposed	85 (92.39)	471,784 (94.36)	Ref			
Predisposed	7 (7.61)	28,216 (5.64)	1.38	0.64-2.98	0.416	
Breed predisposed for pituitary/cortisol-secreting adrenal cancer						**<0.001**
Not predisposed	56 (60.87)	387,621 (77.52)	Ref			
Predisposed	36 (39.13)	112,379 (22.48)	2.22	1.46-3.37	**<0.001**	
Breed predisposed for parathyroid and/or thyroid cancer						0.678
Not predisposed	84 (91.30)	449,812 (89.96)	Ref			
Predisposed	8 (8.70)	50,188 (10.04)	1.17	0.56-2.41	0.678	
Breed predisposed for pituitary/cortisol-secreting adrenal cancer and/or insulinoma						**<0.001**
Not predisposed	50 (54.35)	363,316 (72.66)	Ref			
Predisposed	42 (45.65)	136,684 (27.34)	2.23	1.48-3.36	**<0.001**	
Breed predisposed for parathyroid and/or thyroid cancer and/or insulinoma						0.436
Not predisposed	78 (84.78)	437,174 (87.43)	Ref			
Predisposed	14 (15.22)	62,826 (12.57)	1.25	0.71-2.22	0.436	
Breed predisposed for parathyroid and/or thyroid and/or pituitary/cortisol-secreting adrenal cancer						**0.008**
Not predisposed	55 (59.78)	361,845 (72.37)	Ref			
Predisposed	37 (40.22)	138,155 (27.63)	1.76	1.16-2.67	**0.008**	
Breed predisposed for parathyroid and/or thyroid and/or pituitary/cortisol-secreting adrenal cancer and/or insulinoma						**<0.001**
Not predisposed	49 (53.26)	349,207 (69.84)	Ref			
Predisposed	43 (46.74)	150,793 (30.16)	2.03	1.35-3.06	**<0.001**	
Breed predisposed for reproductive endocrine organ cancer***						0.093
Not predisposed	52 (56.52)	324,602 (64.92)	Ref			
Predisposed	40 (43.48)	175,398 (35.08)	1.42	0.94-2.15	0.093	
Breed predisposed for testicular and/or ovarian cancer						0.226
Not predisposed	86 (93.48)	447,889 (89.58)	Ref			
Predisposed	6 (6.52)	52,111 (10.42)	0.60	0.26-1.37	0.226	
Breed predisposed for one or multiple types of endocrine cancer						**0.006**
Not predisposed	39 (42.39)	283,249 (56.65)	Ref			
Predisposed	53 (57.61)	216,751 (43.35)	1.78	1.17-2.69	**0.006**	

Column percentages are presented in brackets. *CI, confidence interval. **Ref, reference category used as the baseline for comparison with other groups. ***Reproductive endocrine organs include mammary glands, testicles and ovaries. Statistically significant results (*P* < 0.05) are highlighted in bold.

The final breed-focused multivariable logistic regression model included three retained variables: breed, sex/neuter status and age (Table 3). The area under the ROC curve of the final breed-focused model was 0.867, indicating good discrimination (S1 Fig). The model also demonstrated no evidence of poor model fit (Hosmer-Lemeshow test statistic *P* = 0.576). The Soft-Coated Wheaten Terrier, German Pointer and Miniature Schnauzer showed significant breed predispositions for the diagnosis of PCC. Higher odds of PCC diagnosis were found in neutered male dogs compared to entire male dogs and in dogs aged between 9 – < 12 years and 12 – < 15 years compared to dogs aged between 6 – < 9 years. Dogs aged between 0 – < 6 years had lower odds of PCC diagnosis compared to dogs aged between 6 - < 9 years (Table 3).

**Table 3:** Results from the final breed-focused multivariable logistic regression model identifying risk factors associated with phaeochromocytoma diagnosis in dogs under UK primary veterinary care in 2019, as recorded in the VetCompass database.

Variable	Odds ratio	95% CI*	Category *P*-value	Variable *P*-value
Breed				**<0.001**
Crossbreed	Ref**			
Soft-Coated Wheaten Terrier	30.93	7.18-133.22	**<0.001**	
German Pointer	11.01	3.28-36.94	**<0.001**	
Miniature Schnauzer	4.69	1.61-13.63	**0.005**	
Yorkshire Terrier	2.26	0.92-5.59	0.077	
Staffordshire Bull Terrier	1.62	0.72-3.66	0.242	
West Highland White Terrier	1.17	0.35-3.92	0.799	
Lhasa Apso	1.61	0.38-6.83	0.522	
Border Terrier	1.33	0.31-5.66	0.702	
Jack Russell Terrier	1.09	0.46-2.55	0.852	
Shih Tzu	1.46	0.50-4.23	0.490	
English Cocker Spaniel	0.78	0.23-2.60	0.683	
Labrador Retriever	0.67	0.25-1.76	0.414	
Other breeds	0.61	0.34-1.11	0.108	
Sex-neuter				**<0.001**
Female entire	0.47	0.18-1.21	0.117	
Female neutered	0.87	0.45-1.66	0.664	
Male entire	Ref			
Male neutered	2.15	1.22-3.79	**0.009**	
Unrecorded	0	0	0.983	
Age				**<0.001**
0 – < 6	0.07	0.02-0.22	**<0.001**	
6 – < 9	Ref			
9 – < 12	2.40	1.43-4.05	**<0.001**	
12 – < 15	2.42	1.34-4.35	**0.003**	
15+	1.53	0.52-4.47	0.437	
Unrecorded	0	0	**0.985**	

*CI, confidence interval. **Ref, reference category used as the baseline for comparison with other groups. Statistically significant results (*P* < 0.05) are highlighted in bold.

As outlined in the methods, variables derived from or correlated with breed were assessed multivariably by individually substituting them for the *breed* variable in the final breed-focused model. Following this process, nine additional variables were found to be significantly associated with PCC diagnosis: median adult bodyweight, terrier breed status, breed predisposed for mammary gland cancer, breed predisposed for pituitary/cortisol-secreting cancer, breed predisposed for pituitary/cortisol-secreting adrenal cancer and/or insulinoma, breed predisposed for parathyroid, thyroid and pituitary/adrenal cancer, breed predisposed for parathyroid and/or thyroid and/or pituitary/cortisol-secreting adrenal cancer and/or insulinoma, breed predisposed for reproductive endocrine organ cancer and breed predisposed for one or multiple types of endocrine cancer (Table 4). Being a terrier breed showed increased odds for PCC diagnosis (OR 1.65, 95% CI 1.05–2.62) compared to not being a terrier breed, as well as being a breed predisposed for pituitary/cortisol-secreting cancer (OR 1.64, 95% CI 1.08–2.50) and being a breed predisposed for pituitary/cortisol-secreting adrenal cancer and/or insulinoma (OR 1.57, 95% CI 1.04–2.38) compared to not being a breed predisposed for either of these types of endocrine cancer.

**Table 4:** Results for nine breed-derived or breed-associated risk factors that, when substituted for breed in the final breed-focused multivariable logistic regression model, were significantly associated with phaeochromocytoma diagnosis in dogs under UK primary veterinary care in 2019, as recorded in the VetCompass database.

Variable	Odds ratio	95% CI*	Category *P*-value	Variable *P*-value
Terrier				**0.031**
Non-terrier	Ref			
Terrier	1.65	1.05-2.62	**0.031**	
Breed predisposed for mammary gland cancer				0.438
Not predisposed	Ref			
Predisposed	1.18	0.78-1.79	0.438	
Breed predisposed for pituitary/cortisol-secreting adrenal cancer				
Not predisposed	Ref			**0.021**
Predisposed	1.64	1.08-2.50	**0.021**	
Breed predisposed for pituitary/cortisol-secreting adrenal cancer and/or insulinoma				
Not predisposed	Ref			**0.032**
Predisposed	1.57	1.04-2.38	**0.032**	
Breed predisposed for parathyroid, thyroid and pituitary/adrenal cancer				
Not predisposed	Ref			0.213
Predisposed	1.31	0.86-1.98	0.213	
Breed predisposed for parathyroid and/or thyroid and/or pituitary/cortisol-secreting adrenal cancer and/or insulinoma				
Not predisposed	Ref			0.075
Predisposed	1.45	0.96-2.19	0.075	
Breed predisposed for one or multiple types of endocrine cancer				
Not predisposed	Ref			0.317
Predisposed	1.24	0.82-1.87	0.317	

*CI, confidence interval. **Ref, reference category used as the baseline for comparison with other groups. Statistically significant results (*P* < 0.05) are highlighted in bold.

## Discussion

This is the first published study to evaluate the frequency and risk factors for diagnosis of PCC in dogs under primary care veterinary practice in the UK. Novel important breed predispositions were identified, and other risk factors were also recognised which may assist veterinarians in primary care practice with increasing their clinical suspicion of PCC that hopefully promote better patient outcomes with this likely under-diagnosed disease. This study may also help strengthen the value of canine PCC as a model for the human disease and contribute to translational PCC studies.

The annual prevalence of 1 per 100,000 dogs, annual incidence risk of 1 per 100,000 dogs and available-EHR prevalence for the diagnosis of PCC of 4 per 100,000 dogs reported in this study provide new and valuable information on the frequency of PCC diagnosis in dogs attending primary care veterinary practice in the UK. No comparison can be made with other studies as this is the first veterinary study to report these results. Human studies have reported incidence rates of PCC varying from 0.04 per 100,000 to 0.21 per 100,000 person-years, which is between four and a half times to twenty-five times lower than reported for dogs in this study [17–19,54]. However, a minor difference in epidemiological studies of PCC in humans is the inclusion of sympathetic paraganglioma (sPGL) in the reporting of incidence rates. sPGL are extra-adrenal tumours arising from the sympathetic paraganglia which are histologically identical to PCC, share the capacity to synthesise and release catecholamines and have previously been labelled as extra-adrenal PCC, although the World Health Organisation (WHO) consequently reserved the term PCC for intra-adrenal tumours, and even more recently the WHO has defined PCC as intra-adrenal paraganglioma originating from the chromaffin cells of the adrenal medulla [3,8,55–58]. Nevertheless, the majority (80–100%) of the described tumours in human epidemiological studies of PCC/sPGL are PCC, meaning that a strong parallel can still be drawn with this study that does not include sPGL, and suggesting that the incidence rate of intra-adrenal PCC in humans is likely slightly lower than the numbers previously quoted [19]. Additionally, PCC in dogs are clinically different to paraganglioma (PGL) as only PCC are known to overproduce catecholamines with a recent study demonstrating an equal split between adrenergic and noradrenergic phenotypes [59]. The veterinary literature on PGL is limited, therefore PGL were not included in this study [2,60,61].

There are several reasons why primary care veterinary clinicians may be under-diagnosing PCC in dogs. PCC clinical signs are non-specific to the disease and similar clinical signs can occur in many other cardiovascular, neurological or endocrine conditions [5,6,8]. However, clinical risk factors in humans can be used to increase the index of suspicion of PCC, including hyperadrenergic spells, treatment resistant hypertension, and incidentally discovered adrenal masses [62]. Clinical risk factors should also be further researched in dogs to determine if they can be employed alongside demographic risk factors to better identify patients with PCC both in a primary care and in a referral setting. Until recently biochemical assays for diagnosis of PCC such as urinary normetanephrine:creatinine have not been commercially available, which has likely led to fewer cases being detected prior to the start of commercial availability approximately 5 years ago, particularly in primary care practice [22,27,28]. Additionally, access to functional imaging modalities is more limited in primary care practice compared to referral veterinary hospitals [63,64]. In humans, PCC are often incidentally diagnosed during abdominal CT being undertaken for an unrelated condition, although functional imaging with Gallium-68 Dotanoc positron emission tomography computerised tomography (PET-CT) or Metaiodobenzylguanidine scintigraphy (MIBG) combined with single-photon emission computed tomography (SPECT) are preferred imaging modalities if PCC is suspected as they are highly sensitive and specific for the assessment of PCC and its corresponding metastases but are not available for use in veterinary medicine [65–68]. A study describing the epidemiology of human PCC/sPGL in the Netherlands from 1995–2015 found a significant increase of the age-standardised incidence rate coinciding with a reduction in tumour size at the time of initial diagnosis over that twenty-year period and concluded that this was most likely a result of changes in diagnostic practices leading to an earlier detection of these tumours [19]. It could therefore be hypothesised that incidence rates of canine PCC might also increase over time with improved access to veterinary referral hospitals particularly if this is combined with an increased availability of functional imaging, although financial costs for canine pet owners and shorter life expectancies of canines compared to humans may limit such advancements in the veterinary field. Nevertheless, the minimum four and a half times higher incidence rate of PCC in dogs compared to humans combined with a similar frequency of metastasis as well as common interspecies tumourigenic pathways and the possible association of PCC/PGL with chronic hypoxia in brachycephalic breeds as postulated by Holt *et al.* (2014) highlight the value of spontaneous canine PCC as a model for translational studies of human PCC [1,2,8–10,17–19].

Three breeds were identified with predisposition for PCC diagnosis through this study: Soft-Coated Wheaten Terrier, German Pointer and Miniature Schnauzer. All three breeds could be further characterised as being ultra-predisposed, having over four-times higher odds of PCC diagnosis compared to crossbreed dogs [69]. To date, no previous study has reported any breed predispositions for PCC diagnosis, although some previous studies have descriptively flagged the Miniature Schnauzer amongst breeds represented by at least 2 dogs with PCC in their study populations [5,6,33,70–72]. To the authors’ knowledge, Soft-Coated Wheaten Terrier or German Pointer have not previously been suggested at increased risk of PCC diagnosis. The Miniature Schnauzer has previously been reported as predisposed to pituitary adenoma/cortisol-secreting adrenal cancer, and the German Pointer has previously been reported as predisposed for insulinoma and mammary gland cancer but the Soft-Coated Wheaten Terrier has no previously reported predilection for neoplasia [43,45,73]. It is possible that the relative rarity of the Soft-Coated Wheaten Terrier could have prevented previous reporting of a predilection for neoplasia because of underpowering of studies that did not have access to such large denominator populations as the current work. An association was also found between being a terrier breed in general and a diagnosis of PCC, with a similar association also found by Kraai *et al.* (2025) between terrier breed and insulinoma [45]. Terrier breeds constitute one of the twenty-three breed clades that were identified in a large study of canine genomics investigating modern dog breed development and further genetic analysis of these breeds may be warranted to improve our understanding of canine PCC amongst other neoplasia [74].

This study also identified a strong association between sex/neuter status and PCC diagnosis, specifically with male neutered dogs having increased odds of PCC compared to male entire dogs. Interestingly, similar associations between neutering and increased odds of diagnosis of other neoplasia such as osteosarcoma, haemangiosarcoma, and cardiac tumours have previously been reported, although no causation has yet been demonstrated and the retrospective nature of these studies with the potential for selection or confounder biases could lead to an overestimation of the effect of neutering on diagnosis of neoplasia [44,75–78]. Further prospective studies on this topic and research on gonadal hormone influence on the risk of developing neoplasia are warranted. Additionally, neutered dogs tend to be older than their entire counterparts and age was strongly associated with PCC diagnosis, as already reported in previous case series [6]. Dogs aged 9 – < 15 years had increased odds for PCC diagnosis and dogs aged 0 – < 6 years had decreased odds for PCC diagnosis compared to dogs aged 6 – < 9 years which is a typical pattern for a neoplastic condition and also fits the mean age range of ten to twelve years in previous studies and case series [5,6,11]. Age is less likely to be a risk factor specific to PCC and more broadly associated to the diagnosis of a neoplastic disease.

Two associations were found in the final breed-focused model which could have interesting implications in terms of improving the evidence of concurrent endocrine neoplasia (CEN) in veterinary medicine. The current study demonstrated that breeds predisposed for pituitary/cortisol-secreting adrenal cancer as well as breeds predisposed for pituitary/cortisol-secreting adrenal cancer and/or insulinoma had increased odds for PCC diagnosis. This is a finding which is consistent with a previous study of 951 dogs at post-mortem that died or were euthanised at a referral hospital which identified 20 dogs with CEN out of which 7 had PCC and concurrent endocrine neoplasia, with 6/7 having concurrent pituitary adenoma/cortisol-secreting adrenal cancer and 1/7 having concurrent pituitary adenoma and insulinoma [33]. To further support the argument of the existence of a CEN syndrome in dogs with PCC, Barthez *et al.* (1997) also found concurrent pituitary/cortisol-secreting adrenal neoplasia in 13/61 dogs with a PCC diagnosis included in that case series, and more broadly found concurrent neoplasia including a significant proportion of endocrine tumours in 54% of dogs with PCC [6]. Additionally, a case report showed a possible analogy to MEN2 in a Wire-Haired Fox Terrier [79]. However, there is no clear human equivalent for a canine hereditary tumour syndrome where PCC occurs with concurrent pituitary/adrenal tumours or insulinoma. MEN2 and MEN3 are hereditary tumour syndromes which involve up to 24% of human PCC and sPGL, but unlike in dogs the concurrent endocrine neoplasia is usually from the parathyroid or thyroid glands [31,32,80]. More recently, multiple endocrine neoplasia type 4 (MEN4) was discovered in humans which is characterised primarily by parathyroid and pituitary tumours which may occur in association with adrenal, renal and reproductive organ cancer [81,82]. Similarly, a multiple endocrine neoplasia-like syndrome in the rat (MENX) was identified which overlaps MEN1 and MEN2 in humans, with concurrent PCC, pituitary adenomas, parathyroid and thyroid tumours, and is most similar to MEN4 as germline mutations in the tumour suppressor gene *CDKN1B* which codes for the p27 protein is responsible for both MEN4 and MENX [83,84]. It would therefore appear based on current information that if a MEN-like syndrome exists for canine PCC it would not identically match any of the current MEN types but would overlap all of them, whilst perhaps bearing closest resemblance to MEN4. However, the current study’s associations should be interpreted cautiously as it only included PCC diagnoses from the UK whereas the endocrine tumours predisposed breed lists used for this study were not UK-specific and could lack specificity as they relied on prior studies with varying statistical methods.

This study had some limitations. The EHRs were not collected primarily for research purposes and therefore there were issues related to some missing data and also some lack of detail and accuracy in the available clinical records. Some dogs diagnosed with PCC by primary care veterinarians may have been misclassified, potentially representing false positives. Additionally, histopathology on its own does not necessarily provide a definitive diagnosis of PCC if it has not been paired with immunohistochemistry using several markers including chromogranin A, synaptophysin and S1000 that stain chromaffin cells, and without which a different adrenal tumour could be confused for a PCC [2,6,29,85]. Moreover, this study has identified demographic risk factors associated with having PCC rather than risk factors specifically associated with developing this condition. The possibility of a survival bias in the data with cases having been diagnosed with PCC prior to 2019 and surviving up to 2019 may have led to a higher inclusion of cases with a better prognosis and might have consequently missed risk factors associated with more malignant PCC. It is challenging to overcome these limitations in an epidemiological study as PCC is both inherently rare as a diagnosis and challenging to diagnose, particularly in primary care practice. In addition, VetCompass includes only approximately 30% of UK practices which may reduce the ability to generalise to all UK practices, although to mitigate this there is a good geographic distribution of practices of varying sizes and caseloads through the country [86]. Finally, this was an exploratory study meant to support future hypothesis generation and thus did not apply corrections for multiple testing in the various analyses performed, resulting in an increased probability that some of the findings could be statistically significant by chance [87].

In conclusion, this is the first epidemiological study on PCC in the canine population under primary care in veterinary practices in the UK and reported an annual incidence risk of 1 per 100,000 dogs and an annual prevalence of 1 per 100,000 dogs. Several risk factors were identified, in particular: breed, sex/neuter, age, terrier breed, breed predisposed for pituitary adenoma/cortisol-secreting adrenal cancer and breed predisposed for pituitary adenoma/cortisol-secreting adrenal cancer and/or insulinoma. Soft-Coated Wheaten Terrier, German Pointer and Miniature Schnauzer were identified as breeds with ultra-predisposition for PCC diagnosis compared with crossbreeds. These findings can aid veterinarians in primary care practice to improve their recognition of cases with canine PCC and assist future studies on the pathogenesis and genomics of PCC.

## Supporting information

S1 Fig Visualising the discriminatory ability of the final breed-focused model using a receiver operating characteristic (ROC) curve. The area under the ROC curve is 0.867, indicating good discrimination.
(TIF)
