# Supplementary figures and images for: Incidence and risk factors for phaeochromocytoma diagnosis in dogs under primary veterinary care in the UK
Source: PLoS One. 2025 Oct 15;20(10):e0332811. doi: 10.1371/journal.pone.0332811 (PMC12527188; doi:10.1371/journal.pone.0332811)

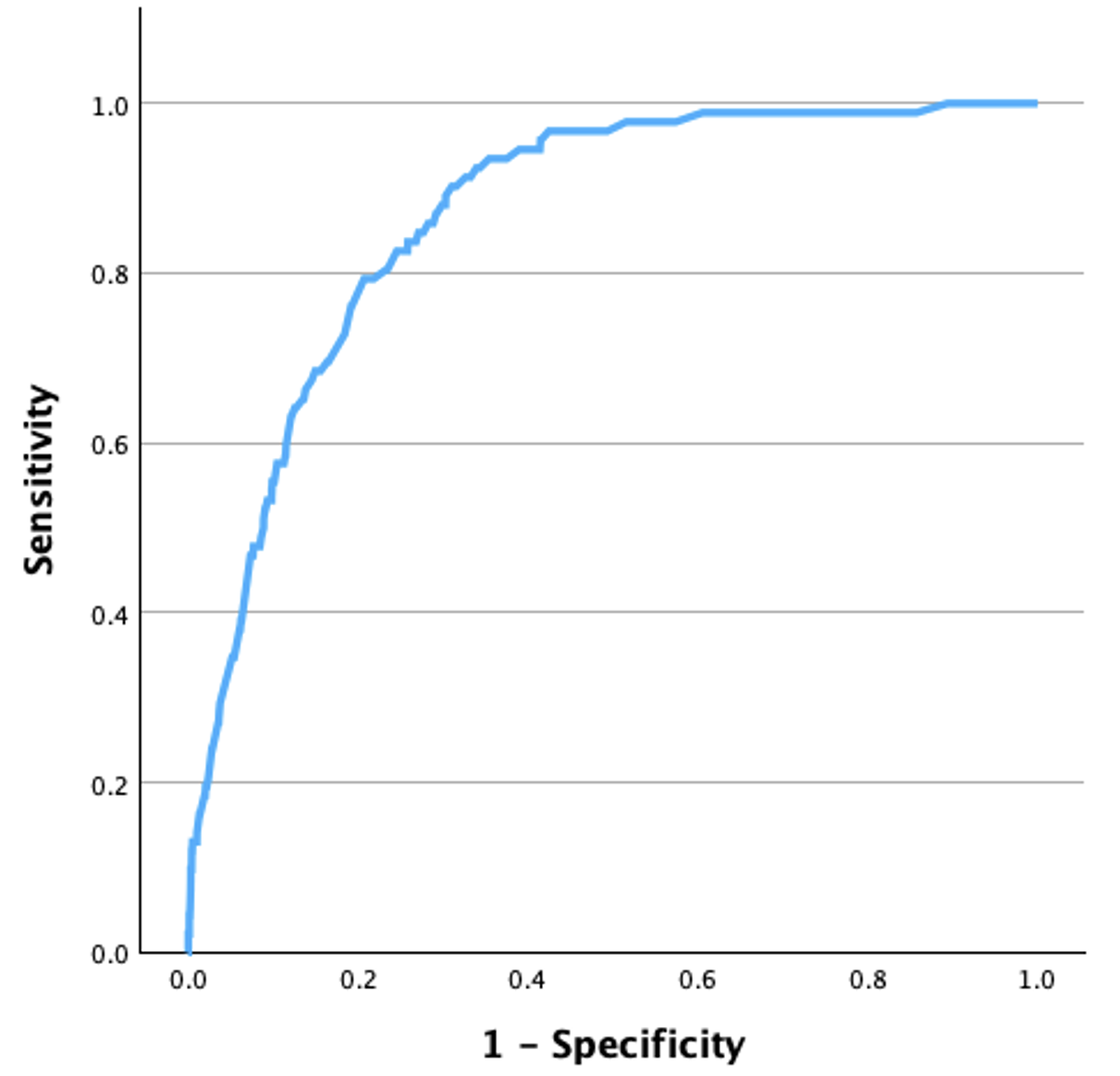

Supplement: S1 Fig — The area under the ROC curve is 0.867, indicating good discrimination. (TIF) [file pone.0332811.s001.tif]
